# Annotations

**Published:** 1909-06-19

**Authors:** 


### Annotations.

### The Tuberculosis Exhibition.

The National Association for the Prevention of Tuberculosis and other Forms of Consumption is to be congratulated on the very sound and practical way in which its scheme of a Tuberculosis Exhibition in Whitechapel has been carried out. For once the reality of some of the factors concerned in the propagation of tuberculosis seems to have been so presented that it impressed the gentlemen of the Press as well as the general public. Indeed, so well have the management advertised their exhibition that its success, were it in the West End, would be assured. The selection of the East End for this educative exhibition, which was opened by Mr. Burns at the Art Gallery in High Street, Whitechapel, is, of course, due to the desire to get in touch with the people who are themselves familiarised by long usage with the unwholesome conditions of life which predispose to tuberculosis; to teach them, if possible, a new way of life, to adapt a phrase of which a good deal has lately been heard. We cannot be so sanguine as to suppose that more than a minute proportion of those whom it is desired to reach will visit the Exhibition, or that many of those who do go will be convinced by what they are shown; but that need not prevent a generous recognition of the Society's objects and labours, which are alike excellent. It is announced that on the conclusion of the Exhibition at Whitechapel it shall make an extended tour of the provinces and of other poor districts of London. No better suggestion could be made, and it is to be hoped that great interest will everywhere be aroused. As a matter of fact, there would be no harm, but perhaps great benefit, in establishing the exhibition for a short time in the West End, for all classes are sadly in need of education in the tuberculosis problem.

### Sanitation in the West Indies.

It will be remembered that Major Ronald Ross has lately expressd profound dissatisfaction with the anti-mosquito and other sanitary crusades in certain of our tropical dependencies which he has visited; and the suggestion was made in these pages (on page 224 of our issue of May 29, 1909) that possibly the distinguished investigator's strictures were but partially deserved. Major Ross singled out Jamaica amongst other colonies in which tropical hygiene is especially backward, an island so near both Panama and Cuba that the example of what American industry can accomplish in those regions renders any slackness particularly unpardonable. It would seem, however, that among the Antilles matters are not nearly so bad. Sir Rubert Boyce writes to the *Times* of June 3 an appreciation of the awakening of the West Indians to the imperative necessity of ridding their islands of mosquito-borne diseases. The education of the rising generation in preventive hygiene is being carefully looked to, and the churches, of all creeds, are co-operating heartily. Is is now in many of our islands a punishable offence to harbour mosquito larvæ on the premises, and 99 summonses have been issued in Barbados alone in the last two months. In fact, Sir Rubert's impressions are favourable all round, and he is convinced that our West Indian possessions have an immense future if the forward progress made towards security of health can be carried out to its proper conclusion. He does not specify Jamaica by name, but it is scarcely to be supposed that he would fail to visit this, the largest and most important of our possessions in those seas. Trinidad, Barbados, St. Lucia, and Antigua seem to be doing best in this matter; but surely Jamaica cannot long lag behind. Between the optimism of Sir Rubert and the pessimism of Major Ross it is perhaps safe to strike a balance.

### Literature and Journalism.

One conclusion arising out of the recent discussion at the Imperial Conference of Editors on the relation of literature to journalism was to the effect that the former might be found even in the columns of a periodical, and is often wanting in matter presented in octavo form. From the nature of the case the verdict may have been biassed: we realise fully that the same objection applies to the present remarks, the sense of which is that in this particular respect medical and lay journalism are precisely comparable. It will, however, be granted that, as regards our leading medical contemporaries in this country, there is a great gulf fixed between the standards of the editorial contributions and of most of the signed articles and papers. English medical journalism ranks high; English medical literature, as a whole, lacks the authority of German, and the lucid expression of French, work. It is not easy to indicate practicable measures for improving matters in the absence of a general raising of the standard of research, and some elevation of the standard of preliminary education demanded of would-be students of medicine. Whilst there can be no possible objection to the former line of progress, any suggestion for increasing the range or severity of the minimum tests of general and academic knowledge which guard the portals of medicine, would arouse considerable opposition from certain practical matter-of-fact persons who hold that a doctor's business is to treat sick people and not to talk cleverly or write cultured English. It is not always the crude efforts of beginners, perhaps passed for publication on the lenient principle of allowing "the first bite," which vitiate records of medical progress. Maiden essays should, and do, receive the reverence due to youth; and anyone can be in the position to quote a paper or two after his name in the Medical Directory, a work which, as recently pointed out in these columns, is largely made up of self-advertisement on the part of its many contributors. There are, unfortunately, seniors who pursue some fashionable line of investigation to the point at which real difficulty is encountered, a point at which scores before them have arrived. Advantage might possibly result from making it compulsory for certain candidates to literary and professional fame to read the full modern literature of some very small subject. Familiarity with notable previous studies should have the same quieting influence as a look through the files at the Patent Office has on an over-confident inventor.

